# 2532: Predictive metabolism studies of varenicline and implications of its metabolites in nicotine addiction

**DOI:** 10.1017/cts.2017.291

**Published:** 2018-05-10

**Authors:** Keeshaloy Thompson, Milton Brown

**Affiliations:** Georgetown - Howard Universities, Washington, DC, USA

## Abstract

OBJECTIVES/SPECIFIC AIMS: The central goal is to predict the metabolites of varenicline and predictively evaluate their propensities for eliciting an increased binding effect in the brain. METHODS/STUDY POPULATION: Molecular modeling computational software and other cheminformatic tools present a strategic in silico strategy to predict a complete metabolic transformation for the varenicline molecule. Molecular docking tools help to highlight key interactions of the varenicline with key metabolizing enzymes that are differentially expressed across a population. This will assist in validating clinical models for smoking cessation. RESULTS/ANTICIPATED RESULTS: Differentialized binding results depending on whatever metabolite is produced. DISCUSSION/SIGNIFICANCE OF IMPACT: Products of metabolism of varenicline may differ in individuals and across groups, thus, binding effects and the propensity for adverse effects may differ in individuals.

